# Covering for the Feet

**Published:** 1875-02

**Authors:** 


### McCOMBER'S PATENT LASTS.

OUR readers will undoubtedly agree with us in saying that their experience thus far has been anything but agreeable, in wearing their useful covering for the feet, which for centuries have been made regardless of principles, which should have been considered of the first importance. Boots and Shoes have been considered from time immemorial as necessary evils, and a part and parcel of our every day belongings; so much so, that we have suffered a thousand pains in wearing what have been sold us, scarcely daring to complain, as all are alike and must necessarily suffer some, if they would have there feet clad at all. But as the longest day has its ending, so is it with the old system of lasting boots and shoes; it has, if not come to an end, met at last its death-blow, and in a few years we shall be able to look back upon the past as a time when the human race travailed in pain, with neither thought nor hope of remedy. Through the persistent efforts of Mr. JOEL McCOMBER, who has studied the subject carefully, a new era has been reached in the manufacture of boots and shoes; and if these are examined carefully, as has been done by scientific men, they will be pronounced by almost all intelligent persons as perfect in their shape, proportions, and fitting. The new last, invented by this gentleman, and recently introduced to public notice, is considered a perfect resemblance to the human foot, being in fact its model, and is therefore derived from a scource which others, in making and shaping lasts, have either overlooked or never thought of. By studying the true proportions and outlines or, in other words, the anatomy of the foot, a perfectly fitting boot and shoe can be made—and this Mr. McComber has done, overcoming the obstacles which have hitherto baffled the last makers. He has perfected the fitting of his lasts and thereby placed, within the reach of all, comfort and enjoyment, where hitherto there has been nothing but pain, vexation, and deformity.

All boots and shoes made after this patent, are handsome and well-fitting at the start, just as a glove fits the hand and requires no breaking in, hence we earnestly and confidently advise persons, of both sexes and of every age, to wear no boots or shoes not made on McComber's last, whom address at 21½ Spruce Street, New York. Descriptive pamphlets will be furnished free to all who apply in person or by letter, as above.

